# DnaK Functions as a Moonlighting Protein on the Surface of *Mycoplasma hyorhinis* Cells

**DOI:** 10.3389/fmicb.2022.842058

**Published:** 2022-03-03

**Authors:** Yao Li, Jia Wang, Beibei Liu, Yanfei Yu, Ting Yuan, Yanna Wei, Yuan Gan, Jia Shao, Guoqing Shao, Zhixin Feng, Zhigang Tu, Qiyan Xiong

**Affiliations:** ^1^School of Life Sciences, Jiangsu University, Zhenjiang, China; ^2^Institute of Veterinary Medicine, Jiangsu Academy of Agricultural Sciences, Key Laboratory of Veterinary Biological Engineering and Technology, Ministry of Agriculture and Rural Affairs, Nanjing, China; ^3^College of Agriculture, Engineering and Science, University of KwaZulu-Natal, Durban, South Africa; ^4^College of Veterinary Medicine, Nanjing Agricultural University, Nanjing, China; ^5^School of Food and Biological Engineering, Jiangsu University, Zhenjiang, China

**Keywords:** *Mycoplasma hyorhinis*, DnaK, adhesion, plasminogen, extracellular matrix, moonlighting protein, virulence factor

## Abstract

*Mycoplasma hyorhinis* is a common pathogen of swine and is also associated with various human tumors. It causes systemic inflammation, typically polyserositis and polyarthritis, in some infected pigs. However, the pathogenic mechanism of *M. hyorhinis* remains unclear. DnaK is a highly conserved protein belonging to the heat-shock protein 70 family of molecular chaperones, which plays important roles as a moonlighting protein in various bacteria. In the present study, we identified the surface exposure of *M. hyorhinis* DnaK. Two virulent strains expressed more DnaK on their surface than the avirulent strain. Thereafter, the potential moonlighting functions of DnaK were investigated. Recombinant *M. hyorhinis* DnaK (rMhr-DnaK) was found to be able to adhere to swine PK-15 cells and human NCI-H292 cells. It also bound to four extracellular matrix components—fibronectin, laminin, type IV collagen, and vitronectin—in a dose-dependent manner. ELISA demonstrated an interaction between rMhr-DnaK and plasminogen, which was significantly inhibited by a lysine analog, ε-aminocaproic acid. rMhr-DnaK-bound plasminogen was activated by tissue-type plasminogen activator (tPA), and the addition of rMhr-DnaK significantly enhanced the activation. Finally, a DnaK-specific antibody was detected in the serum of pigs immunized with inactivated vaccines, which indicated good immunogenicity of it. In summary, our findings imply that DnaK is an important multifunctional moonlighting protein in *M. hyorhinis* and likely participates extensively in the infection and pathogenesis processes of *M. hyorhinis*.

## Introduction

*Mycoplasma hyorhinis* is a species of mycoplasmas (class Mollicutes), which are small-sized, cell-wall-free, prokaryotic organisms. *M. hyorhinis* was recognized as a pathogen of swine in Carter and McKay (1953). It was once considered to be a harmless commensal bacteria colonizing the tonsils and respiratory tract epithelium; however, its pathogenicity was subsequently identified and confirmed. It is well recognized now as a cause of polyserositis and polyathritis primarily in nursery-age pigs. It has occasionally also been linked with pneumonia, eustachitis, otitis, conjunctivitis, meningoencephalitis, and abortion in pigs (Zimmerman et al., 2019). The disease leads to reduced performance or culling of affected animals, which results in economic losses to the pig production industry (Clavijo et al., 2017; Martinson et al., 2018). *M. hyorhinis* also infects human beings. Although it seems not to be a commensal bacteria wildly popular in human, but the infection proportion of *M. hyorhinis* has been reported to be significantly higher in cancer tissues than the tissues of patients without cancer (Huang et al., 2001; Vande Voorde et al., 2014a). *M. hyorhinis* induces cancer cell migration and invasion *in vitro* and metastasis *in vivo* (Yang et al., 2010). Related mechanisms may include NF-κB signaling pathway activation (Duan et al., 2014a), increased activity of MMP-2 (Gong et al., 2008), enhanced phosphorylation of EGFR and ERK1/2 (Duan et al., 2014b), and IL-6-mediated STAT3 signaling activation (Gomersall et al., 2015). *M. hyorhinis* infection also increases the resistance of tumor cells to anticancer agents. In *M. hyorhinis*-infected tumor cell cultures, mycoplasma-encoded cytidine deaminase and pyrimidine nucleoside phosphorylase were shown to compromise the antitumor activity of nucleoside analogs (Vande Voorde et al., 2014b). Multidrug resistance (MDR) to various chemotherapeutic agents has been observed in *M. hyorhinis*-infected hepatocarcinoma cells, which depends on the interaction between a surface lipoprotein P37 of *M. hyorhinis* and annexin A2 of the cell (Liu et al., 2017).

The knowledge on the pathogenic mechanism of *M. hyorhinis* remains extremely limited. It is assumed that *M. hyorhinis* binds to the ciliated respiratory epithelium *via* adhesion molecules such as variable lipoprotein family (Vlp) (Xiong et al., 2016), tumor-associated lipoprotein P37 (Duan et al., 2014a), and glyceraldehyde-3-phosphate dehydrogenase (GAPDH) (Wang et al., 2021). Colonized *M. hyorhinis* pass through the epithelial barrier, leading to systemic dissemination, and develop diseases in multiple tissues and organisms. The epithelial damage caused by other pathogens (Chen et al., 2016; Lee et al., 2016) and the interaction between *M. hyorhinis* and host plasminogen/plasmin system (Wang et al., 2021) may contribute to its systemic spread. Phase and size variation of Vlp results in highly frequent changes in the surface antigenicity of *M. hyorhinis*, which allows it to escape host immune recognition (Citti et al., 2000). An interaction between *M. hyorhinis* and factor H and its function in escaping complement killing has also been implicated (Yu et al., 2020).

Notably, *M. hyorhinis* proteins that have been identified to date do not have only one function. Vlp functions in immune escape (Yogev et al., 1991; Citti et al., 2000) and cytoadhesion (Xiong et al., 2016); P37 participates in cancer promotion (Gong et al., 2008; Kim M. K. et al., 2019), drug resistance induction (Liu et al., 2017), and cytoadhesion (Duan et al., 2014a); and GAPDH functions in glycolysis, cytoadhesion and plasminogen/plasmin system hijacking (Wang et al., 2021). Proteins that are associated with more than one clearly distinct biological activity are named moonlighting proteins (Henderson and Martin, 2013). Moonlighting proteins are very common in mycoplasmas because they should utilize their small genomes efficiently (Grundel et al., 2016; Yu et al., 2018). Many molecular chaperones in bacteria are recognized as moonlighting proteins (Jeffery C. J., 2018). DnaK is a highly conserved protein belonging to the heat-shock protein (HSP) 70 family of molecular chaperones (Mayer et al., 2000; Perales-Calvo et al., 2018). The primary function of DnaK is helping unfolded or partially folded proteins to achieve their proper functional conformation. It also participates in the assembly of large multi-protein complexes, preventing the formation and precipitation of unstable protein aggregates. DnaK has been reported to moonlight in *M. pneumoniae* (Hagemann et al., 2017). The DnaK protein of *M. fermentans* has been shown to have broad oncogenic properties (Zella et al., 2018). However, the role of DnaK in *M. hyorhinis* infection remains unclear. In the present study, the surface exposure of *M. hyorhinis* DnaK was determined. Significant higher expression on the surface of virulent strains was observed, indicating the possibility of DnaK as a virulence factor of *M. hyorhinis*. Its potential moonlighting functions in interacting with host cells and molecules were further investigated.

## Materials and Methods

### Mycoplasma Strains and Cell Lines

*Mycoplasma hyorhinis* strains HEF-16 and JS-15 were isolated from pigs showing typical clinical signs of *M. hyorhinis* infection and were able to reproduce the disease in challenge test (Supplementary Figure S1). *M. hyorhinis* strain HUB-1, kindly provided by Prof. Shaobo Xiao from Huazhong Agricultural University, China, was unable to induce any disease in a challenge test (Supplementary Figure S1). Pig kidney cell line PK-15 (ATCC, Manassas, VA, United States, CCL-33) and human airway epithelial cell line NCI-H292 (ATCC, Manassas, VA, United States, CRL-1848) were cultured in Dulbecco’s modified Eagle medium (DMEM) and RPMI 1640 (Thermofisher Scientific, Waltham, MA, United States), respectively, supplemented with 10% fetal bovine serum (FBS). The cells were grown at 37°C in humidified air with provision of 5% CO_2_.

### Protein Expression and Purification

The gene encoding *M. hyorhinis* DnaK (NCBI accession number: CP002170) was synthesized by GenScript Biotech Corp, Nanjing, China. The sequence was optimized with *E. coli*-preferred codons, and two TGA codons (coding Trp in mycoplasmas) were mutated into TGG codons. The gene was inserted into the pET-32a (+) vector (GenScript, Nanjing, China) between *Eco*RI and *Hind*III restriction sites to generate recombinant plasmid pET-32a-rMhr-DnaK that express a Trx-fusion protein. The plasmid was transformed into the *E. coli* strain BL21 (DE3). The bacterial cells were cultured in Luria–Bertani (LB) medium at 37°C. When the OD_600*nm*_ values of the bacterial cultures reached approximately 0.8, isopropyl-beta-D-thiogalactopyranoside (IPTG) at a final concentration of 1 mM was added to induce protein expression and incubated at 18°C overnight. The bacterial cells were collected by centrifugation at 12,000 × *g* for 10 min and resuspended in Tris–HCl buffer (pH = 8.0) with 300 mM NaCl and 20 mM imidazole. Then, the cells were lysed by sonication at 4°C, and the cell pellets were removed by centrifugation at 10,000 × *g* for 30 min. Supernatants was incubated with Ni Sepharose 6 FF resin for 1 h at 4°C, and the target protein was eluted with 150 mM imidazole. Purified protein was confirmed by SDS-PAGE and named as rMhr-DnaK.

### Preparation of Polyclonal Antibody

Polyclonal antibody against the recombinant protein rMhr-DnaK was obtained by immunizing 4-week-old New Zealand white rabbits. Purified rMhr-DnaK was emulsified with complete (the first immunization) and incomplete (the second and third immunization) Freund’s adjuvant (1:1, v/v). Rabbits were subcutaneously immunized with 1 mg of recombinant protein three times at 2-week intervals. Antisera were collected 2 weeks after the third immunization and antibody titer was detected by ELISA. The blood was collected from heart when the titer was higher than 1,00,000, and antisera was extracted and store at −20°C until use.

The ability of the prepared polyclonal antibody to recognize DnaK from *M. hyorhinis* and rMhr-DnaK was determined by Western blotting. The whole cell lysate of *M. hyorhinis* and the purified rMhr-DnaK protein were subjected to 12% SDS-PAGE and transferred to a polyvinylidene fluoride (PVDF) membrane. After blocking with 5% skim milk in Tris-buffered saline with Tween 20 (TBST) buffer for 1 h at 37°C, the membrane was incubated with the anti-DnaK serum (1:1,000 dilution) overnight at 4°C, followed by horseradish peroxidase (HRP)-conjugated goat anti-rabbit IgG (1:10,000 dilution; Boster, China) for 1 h at 37°C. Finally, filters were developed with Electro-Chemi-Luminescence (ECL) substrate using a ChemiDoc XRS + system (Bio-Rad, Hercules, CA, United States). Preimmune serum was used as a negative control instead of anti-DnaK serum.

### Detection of Surface-Exposed DnaK in *Mycoplasma hyorhinis* Cells

The surface localization of DnaK in *M. hyorhinis* (strain HEF-16) was first investigated by colony blotting with DnaK-specific antisera under conditions that would not damage the cell membrane of *M. hyorhinis* (Supplementary Table S1). PVDF membranes were gently placed on mycoplasma colonies on the surface of agar plates. After 5 min, filters were removed, blocked for 1 h at 37°C with Tris-buffered saline (TBS) containing 5% skim milk, and incubated overnight at 4°C in TBS containing 5% skim milk and anti-DnaK serum (1:1,000 dilution). Filters were washed four times with TBS with an interval of 15 min and treated with HRP-conjugated goat anti-rabbit IgG (1:10,000 dilution; Boster, China) for 1 h at 37°C. Finally, filters were developed with ECL substrate using the ChemiDoc XRS + system. Preimmune serum was used as a negative control instead of anti-DnaK serum. Antiserum against GAPDH whose surface expression has been identified previously (Wang et al., 2021) was used as a positive control.

Subsequently, flow cytometry (FACS) analysis was used to confirm the surface localization of DnaK and analyze the difference between strains with different virulence. In brief, *M. hyorhinis* cultures of strains HEF-16, JS-15, and HUB-1 (10^8^ color change units (CCU)/mL) were centrifuged at 15,000 × *g* for 20 min at 4°C, respectively. After blocking with phosphate-buffered saline (PBS) containing 1% bovine serum albumin (BSA), *M. hyorhinis* cells in the precipitate were collected and incubated with anti-DnaK serum or anti-GAPDH serum at a 1:100 dilution in PBS for 1 h at 37°C. *M. hyorhinis* incubated with preimmune rabbit serum was used as negative control. After washing with PBS, *M. hyorhinis* cells were stained with fluorescein isothiocyanate (FITC)-conjugated goat anti-rabbit IgG at a 1:500 dilution (Boster, China) for 1 h at 37°C. The fluorescence intensity was detected using a flow cytometer (BD Accuri^®^ C6). The mean fluorescence intensity (MFI) of *M. hyorhinis* incubated with anti-DnaK serum or anti-GAPDH serum was expressed as the percentage of that of *M. hyorhinis* incubated with preimmune serum.

### Adhesion Inhibition of *Mycoplasma hyorhinis* to Cells by Anti-DnaK Polyclonal Antibody

*Mycoplasma hyorhinis* cells (1 × 10^7^ CCU/mL) were washed three times with PBS and pre-incubated with anti-DnaK serum or preimmune serum (1:20 dilution) at 37°C for 30 min. Bacteria suspended in cell medium were added to 24-well cell plates containing confluent PK-15 or NCI-H292 cells and incubated at 37°C for 6 h. After washing with PBS to remove non-adherent mycoplasmas, the cells were digested with 0.25% trypsin at 37°C for 5 min, and the amount of mycoplasma were determined by quantitative real-time PCR (Fourour et al., 2018).

### Adhesion of rMhr-DnaK to Host Cells

An indirect immunofluorescence assay was used to determine whether the rMhr-DnaK protein adhered to cell surface. The 1 × 10^5^/mL cells were propagated in a 96-well cell culture dish for 24 h. The original medium was removed, and 100 μL of rMhr-DnaK in medium (200 μg/mL) was added and incubated at 37°C for 2 h. Cells incubated with the recombinant protein purified by nickel column affinity chromatography from *E. coli* containing the empty pET-32a (+) vector were used as negative control. Cells incubated with the recombinant rMhr-GAPDH protein were used as positive control. The unbound proteins were washed with PBS. Then, the cells were fixed with cold ethanol for 30 min at 4°C and blocked with PBS containing 5% BSA for 1 h at 37°C. The adherence was assessed by rabbit anti-DnaK serum (1:500 dilution) or rabbit anti-GAPDH serum (1:500 dilution) as primary antibodies and FITC-labeled goat anti-rabbit IgG (1:500 dilution; Boster, China) as secondary antibody. Finally, cell nuclei were stained with DAPI, and the immunofluorescence was detected using a fluorescence microscope (Zeiss, Jena, Germany).

### Binding of rMhr-DnaK to Cell Membrane Proteins

The cell membrane proteins of PK-15 and NCI-H292 cells were prepared by a commercial Membrane and Cytosol Protein Extraction Kit according to the manufacturers’ instructions (Tiangen Biotech, China). The ability of rMhr-DnaK to bind cell membrane proteins was quantitatively determined by a microtiter plate adhesion assay (MPAA) (Xiong et al., 2016). In brief, a 96-well ELISA plate was coated with 100 μL cell membrane proteins (10 μg/mL) overnight at 4°C. After blocking with 5% BSA, the plate was incubated for 2 h at 37°C with 100 μL of rMhr-DnaK solution at different concentrations (ranging from 1.56 to 100 μg/mL) or PBS. Unbound proteins were removed by washing with PBS Tween (PBST), and the adherence was evaluated by adding 100 μL of mouse anti-Trx-tag monoclonal antibody (1:1,000 dilution; Sangon, China) followed by 100 μL of HRP-conjugated goat anti-mouse IgG (1:10,000 dilution; Boster, China). After washing, the substrate containing 3,3′,5,5′-tetramethylbenzidine and H_2_O_2_-urea was added, and the plates were incubated at 37°C for 15 min. Then, 2 M H_2_SO_4_ was added to stop the reaction, and the optical density (OD) of the solution was measured at 450 nm. For the adherence inhibition assay, 50 μg/mL of rMhr-DnaK was mixed with anti-DnaK or preimmune serum at various dilutions (1:10, 1:25, and 1:50) and added into the microtiter plate.

### Binding of rMhr-DnaK to Different Extracellular Matrix Components

ELISA plate was coated with 100 μL of 3 μg/mL fibronectin (Sigma-Aldrich, Burlington, MA, United States, 10838039001), collagen type IV (Sigma-Aldrich, Burlington, MA, United States, C7521), laminin (Sigma-Aldrich, Burlington, MA, United States, L2020), or vitronectin (Sigma-Aldrich, Burlington, MA, United States, 5051) overnight at 4°C. After blocking with 5% BSA, 100 μL of rMhr-DnaK or BSA solutions at different concentrations (ranging from 1.56 to 100 μg/mL) or PBS was added. After washing with PBST, the adherence was evaluated by adding 100 μL of mouse anti-Trx-tag monoclonal antibody (1:1,000 dilution; Sangon, China) followed by 100 μL of HRP-conjugated goat anti-mouse IgG (1:10,000 dilution; Boster, China). After washing, the substrate was added, and the absorbance was measured at 450 nm.

### Interaction Between rMhr-DnaK and Host Plasminogen

The ability of rMhr-DnaK to bind plasminogen was first detected. An ELISA plate was coated with 100 μL of 3 μg/mL plasminogen (Sigma-Aldrich, Burlington, MA, United States, SRP6518). After blocking, 100 μL of rMhr-DnaK or BSA solutions at different concentrations (ranging from 1.56 to 100 μg/mL) or PBS was added. The binding of rMhr-DnaK to plasminogen was determined using the method described in the section “Binding of rMhr-DnaK to Cell Membrane Proteins.”

To determine the function of lysine residues of DnaK in its interaction with plasminogen, the lysine analog ε-aminocaproic acid (ε-ACA, Sigma-Aldrich, Burlington, MA, United States) was added as a competitive inhibitor. ELISA plates were coated with 100 μL rMhr-DnaK solution (30 μg/mL) overnight at 4°C. After blocking, the plate was incubated for 2 h at 37°C with 100 μL of 5 μg/mL plasminogen in the presence or absence of increasing concentrations (100, 200, 400, 800, and 1,600 mM) of ε-ACA. After washing, bound plasminogen was detected by adding rabbit anti-plasminogen polyclonal antibody (1:2,000 dilution; Boster, China) followed by HRP-conjugated goat anti-rabbit IgG (1:10,000 dilution). After washing, the substrate was added, and the absorbance was measured at 450 nm.

The effect of rMhr-DnaK binding on the activation of plasminogen was assessed. An ELISA plate was coated with 100 μL of 10 μg/mL rMhr-DnaK overnight at 4°C. After blocking with 5% BSA, the plate was incubated for 2 h at 37°C with 100 μL of 5 μg/mL plasminogen. After washing with PBS, 100 μL of tissue-type plasminogen activator (tPA; 200 ng/mL, Sigma-Aldrich, Burlington, MA, United States) was added, and the plate was incubated for 2 h at 37°C. After washing, 100 μL of the plasmin-specific substrate D-valyl-leucyl-lysine-p-nitroanilide dihydrochloride (Sigma-Aldrich, Burlington, MA, United States) was added at a final concentration of 0.4 mM. Plates were incubated overnight at 37°C, and absorbance was detected at 405 nm. BSA-coated wells were used as the negative control. Wells without tPA were set to determine whether the recombinant protein activated the plasminogen by itself.

The activation was further determined under a solution state. rMhr-DnaK (20 μg/mL) and plasminogen (20 μg/mL) were mixed and incubated at 37°C for 1 h and then added into a flat-bottomed 96-well ELISA plate. Then, tPA was added to a concentration of 500 ng/mL. After 15 min incubation, 0.4 mM of substrate was added, and the plate were incubated at 37°C. OD_405*nm*_ was measured every 15 min for 120 min. Wells containing only plasminogen with tPA or rMhr-DnaK with plasminogen or rMhr-DnaK with tPA were used as controls.

### Detection of the DnaK-Specific Antibody in *Mycoplasma hyorhinis*-Immunized Pigs

Fresh *M. hyorhinis* cultures were inactivated with 0.01% formaldehyde at 37°C for 24 h and mixed with Tween-80 (4%, v/v). Vaccines were made by emulsifying the aqueous phase with the Marcol white mineral oil adjuvant at 10:25 (v/v). Six-week-old Bama miniature pigs originally developed from caesarian-derived colostrum-deprived (CDCD) pigs were obtained from a pig farm. Six pigs, confirmed to be free from colonization by *M. hyorhinis* by nested PCR on nasal swabs, were randomly separated into two groups. Animals in group 1 were intramuscularly inoculated with 2 mL of *M. hyorhinis* inactivated vaccine (10^9^ CCU/mL), and the inoculation was repeated 14 days later. Animals in group 2 were not immunized. Serum samples were collected every 14 days until 42 days after the first immunization and assayed for antibody against DnaK. In brief, ELISA plates were coated with the rMhr-DnaK protein (10 μg/mL) overnight at 4°C. After blocking with 5% BSA, each well was incubated for 30 min at 37°C with 100 μL of serum sample diluted in PBS containing 5% BSA at 1:100, followed by 100 μL HRP-conjugated goat anti-swine IgG (Bethyl Laboratories, Montgomery, United States) at a dilution of 1:10,000. After washing, the substrate was added, and the absorbance was measured at 450 nm.

### Statistical Analysis

Data are expressed as mean ± SD. Statistical analysis was performed using GraphPad Prism software (Version 8.2.1). Independent samples *t*-test was used to analyze the data difference of FACS between specific antiserum and preimmune serum, the data in antibody-mediated adherence inhibition assay, the data difference between rMhr-DnaK and BSA in ECM and plasminogen binding assay. Repeated measures ANOVA was used to analyze the dynamic changes of plasminogen activation and anti-DnaK antibody production. The statistical analysis on other data was performed using one-way analysis of variance (ANOVA) followed by Dunnett’s multiple comparisons test. *P*-values < 0.05 were considered statistically significant.

## Results

### Expression and Purification of rMhr-DnaK and Polyclonal Antibody Production

**FIGURE 1:**
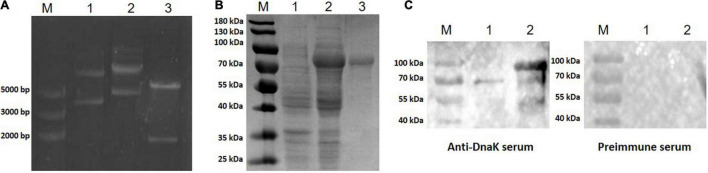
Construction, expression, and purification of the recombinant protein rMhr-DnaK. **(A)** Identification of the recombinant plasmid. M, DNA marker; lane 1, empty pET-32a(+) vector; lane 2, pET-32a-rMhr-DnaK; and lane 3, pET-32a-rMhr-DnaK digested by *Eco*RI and *Hind*III. **(B)** Expression and purification of the recombinant protein rMhr-DnaK. M, protein molecular weight marker; lane 1 and 2, whole cell lysate of *E. coli* BL21 carrying the recombinant plasmid pET-32a-rMhr-DnaK before and after induction by IPTG, respectively; lane 3, purified rMhr-DnaK. **(C)** Specific binding of the anti-DnaK polyclonal antibody to DnaK detected by Western blotting. M, protein molecular weight marker; lane 1, whole cell lysate of *Mycoplasma hyorhinis*; lane 2, purified rMhr-DnaK. Preimmune serum served as negative control.

The Full-length *DnaK* gene was designed according to the amino acid sequence of DnaK of *M. hyorhinis* strain HUB-1 and optimized according to *E. coli* codon usage. The synthetic gene was inserted into the expression vector pET-32a (+) (Figure 1A) and transformed into *E. coli*. The correct sequence was verified by DNA sequencing, and the recombinant engineering strain was obtained and named as pET-32a-rMhr-DnaK. IPTG was added to the *E. coli* cultures to induce the expression of recombinant protein. The SDS-PAGE results showed a distinct band between 70 and 100 kDa (Figure 1B, Lane 2), which was consistent with the theoretical molecular weight (79.8 kDa) of the recombinant protein rMhr-DnaK. After cell lysis, the recombinant protein in the supernatant was purified by nickel column affinity chromatography (Figure 1B, Lane 3). Rabbit anti-DnaK polyclonal antibody was prepared by immunizing the purified rMhr-DnaK protein. The serum was collected after three immunizations, and the antibody titer was detected by ELISA to be 1:128,000. The antibody could specifically react to the DnaK from *M. hyorhinis* and purified rMhr-DnaK protein (Figure 1C).

### Expression of DnaK on the Surface of *Mycoplasma hyorhinis*

**FIGURE 2:**
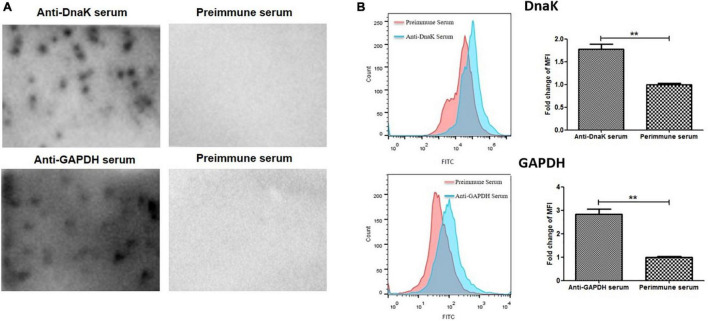
Surface expression of DnaK. **(A)** Detection of *M. hyorhinis* DnaK surface display by colony blot. *M. hyorhinis* colonies (strain HEF-16) on the surface of agar plates were transferred to PVDF membranes and detected by anti-DnaK serum, anti-GAPDH serum or their preimmune serum, respectively. **(B)** Detection of *M. hyorhinis* DnaK surface display by FACS. *M. hyorhinis* strain HEF-16 cells were incubated with anti-DnaK serum, anti-GAPDH serum or their preimmune serum, respectively. The level of MFI of *M. hyorhinis* incubated with anti-DnaK serum or anti-GAPDH serum is expressed as the percentage of that of *M. hyorhinis* incubated with preimmune serum. Data are the mean ± SD of samples in triplicate. **P < 0.01.

**FIGURE 3:**
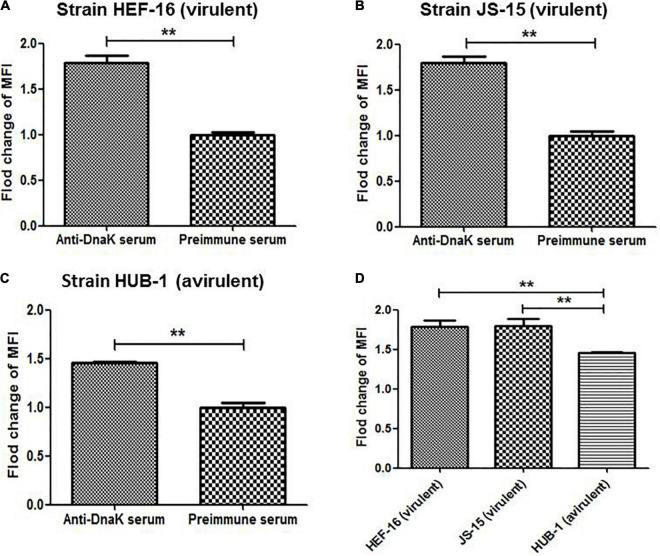
Surface expression of DnaK in different *M. hyorhinis* strains. Two virulent strains HEF-16 **(A)** and JS-15 **(B)** and one avirulent stain HUB-1 **(C)** were incubated with anti-DnaK serum or preimmune serum. The level of MFI of *M. hyorhinis* incubated with anti-DnaK serum is expressed as the percentage of that of *M. hyorhinis* incubated with preimmune serum. The surface expression of DnaK in three different strains was compared **(D)**. Data are the mean ± SD of samples in triplicate. **P < 0.01.

Surface localization of DnaK in *M. hyorhinis* was investigated by detecting the binding of anti-DnaK antibody to bacterial surface under conditions that did not damage the cell membrane of *M. hyorhinis.* First, colony blotting was carried out. As a result, significant signals were demonstrated after incubation of the PVDF membranes with the anti-DnaK serum or anti-GAPDH serum but not after its incubation with preimmune serum (Figure 2A). Thereafter, the surface expression of DnaK was further examined by FACS analysis. As shown in Figure 2B, significantly higher MFI of *M. hyorhinis* incubated with anti-DnaK serum or anti-GAPDH serum were observed than that of *M. hyorhinis* incubated with the preimmune serum (*P* < 0.01). Three strains of *M. hyorhinis* with different virulence were compared for their surface expression of DnaK. As shown in Figure 3, the virulent strains HEF-16 and JS-15 showed higher MFI than the strain HUB-1 which lacked the ability to cause clinical disease (*P* < 0.01).

### Inhibition of Anti-DnaK Serum on Adhesion of *Mycoplasma hyorhinis* to Host Cells

**FIGURE 4:**
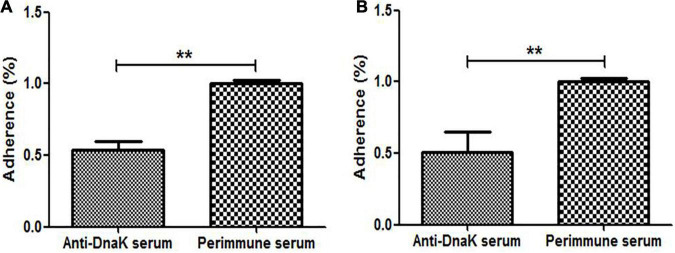
Adhesion Inhibition of *M. hyorhinis* to cells by anti-DnaK polyclonal antibody. *M. hyorhinis* cells pre-incubated with anti-DnaK serum or preimmune serum were added to PK-15 **(A)** or NCI-H292 **(B)** cells. The amount of bound *M. hyorhinis* were determined by qPCR. Adhesion rate: number of mycoplasmas in the group incubated with anti-DnaK serum/number of mycoplasmas in the group incubated with preimmune serum. Data are the mean ± SD of samples in triplicate. **P < 0.01.

Antibody inhibition assay was performed to investigate the function of surface-exposed DnaK in cytoadhesion of *M. hyorhinis*. The number of *M. hyorhinis* attached to swine-origin PK-15 cells (Figure 4A) and human-origin NCI-H292 cells (Figure 4B) were greatly reduced after being incubated with anti-DnaK serum, compared with those of the *M. hyorhinis* incubated with preimmune serum (*P* < 0.01). It was indicated that surface-localized DnaK plays a role in the adherence of *M. hyorhinis* to host cells.

### Adhesion of rMhr-DnaK to Host Cells

**FIGURE 5:**
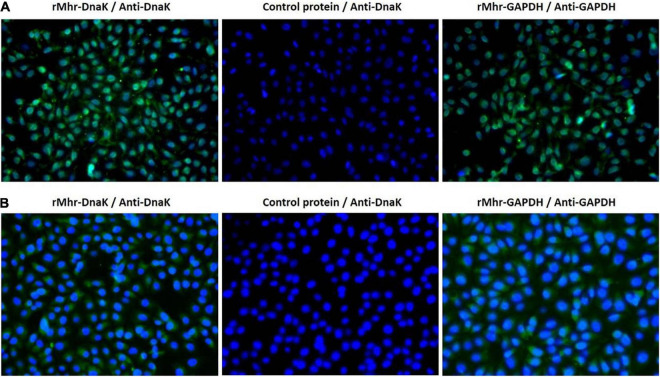
Adherence of rMhr-DnaK to host cells detected by indirect immunofluorescence assay. PK-15 **(A)** and NCI-H292 **(B)** cells were incubated with rMhr-DnaK, rMhr-GAPDH or control protein pET32a. Bound proteins were detected by antisera of rabbits immunized with rMhr-DnaK or rMhr-GAPDH and fluorescein isothiocyanate-labeled secondary antibody (green). Cell nuclei were stained with DAPI (blue).

**FIGURE 6:**
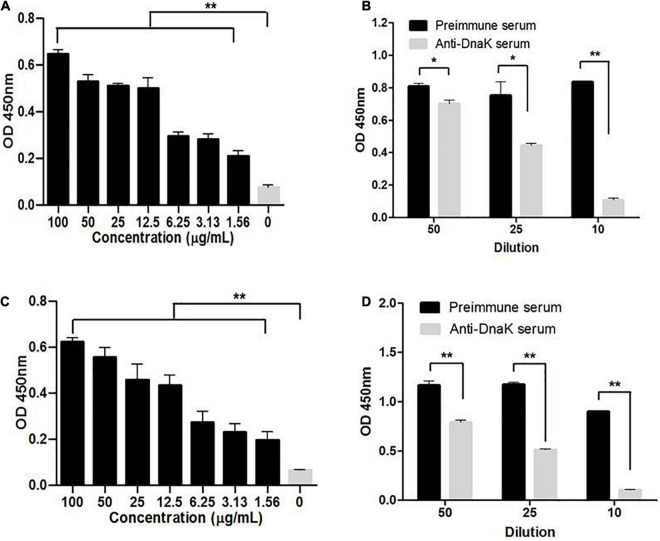
Ability of rMhr-DnaK to bind cell membrane proteins. Microtiter plates were coated with the extracted membrane proteins of PK-15 **(A)** or NCI-H292 **(B)** cells. Different concentrations of the rMhr-DnaK protein or PBS were added to individual wells. Bound proteins were detected using mouse anti-Trx-tag monoclonal antibody. The inhibition of anti-DnaK serum on the binding of rMhr-DnaK to PK-15 **(C)** or NCI-H292 **(D)** cell membrane proteins was determined. Preimmune serum served as negative control. The tests were performed in three independent experiments. Data are the mean ± SD of triplicate wells from a representative of three independent experiments. *P < 0.05 and **P < 0.01.

The role of DnaK as *M. hyorhinis* adhesin was further investigated by determining its ability to adhere to host cells. The rMhr-DnaK protein was added to the cultures of PK-15 and NCI-H292 cells, and the adhered recombinant protein was detected after 2 h incubation by indirect immunofluorescence method. The results are shown in Figure 5. Distinct green fluorescence signals were observed in the wells of both kinds of cells incubated with rMhr-DnaK or rMhr-GAPDH, which was distributed in the whole cell-covered area. No green fluorescence signal was observed in the intercellular space. The wells with control protein had no positive fluorescence signal. Furthermore, the binding of rMhr-DnaK to cell membrane proteins was quantitatively detected by MPAA. The results showed that rMhr-DnaK significantly bound to the membrane proteins of both the PK-15 and NCI-H292 cells (*P* < 0.01). With the increase in rMhr-DnaK concentration, the OD value gradually increased in a dose-dependent manner (Figures 6A,B). The binding was significantly inhibited by anti-DnaK serum, compared to the preimmune serum (Figures 6C,D).

### Binding Ability of rMhr-DnaK to Extracellular Matrix Components

**FIGURE 7:**
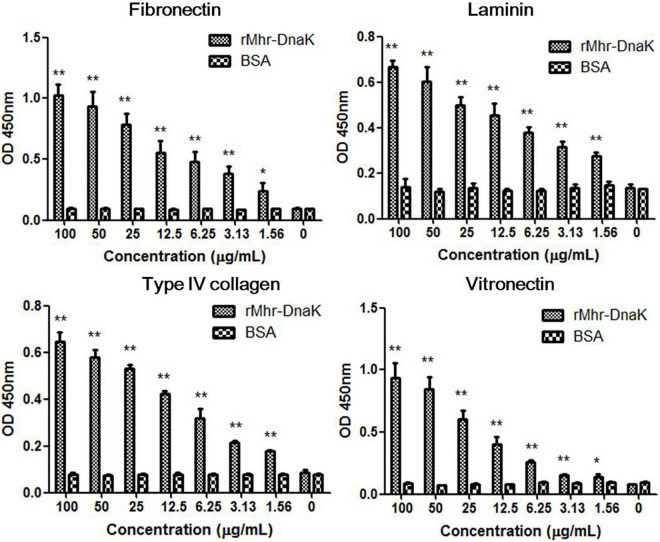
Binding of rMhr-DnaK to different extracellular matrix (ECM) components. ELISA plates were coated with ECM components, including fibronectin, type IV collagen, laminin, and vitronectin. Different concentrations of the rMhr-DnaK protein, BSA, or PBS were added to individual wells. Bound proteins were detected using mouse anti-Trx-tag monoclonal antibody. The assays were performed in three independent experiments. Data are the mean ± SD of triplicate wells from a representative of three independent experiments. *P < 0.05 and **P < 0.01, compared with the wells containing BSA instead of rMhr-DnaK proteins.

The binding of rMhr-DnaK to extracellular matrix (ECM) was assessed by detecting the adherence of rMhr-DnaK to the 96-well ELISA plate coated with different ECM components. As shown in Figure 7, the recombinant protein significantly bound to all the four different ECM components, fibronectin, type IV collagen, laminin and vitronectin, in a dose-dependent manner (*P* < 0.01, compared with BSA).

### Interaction Between rMhr-DnaK and Host Plasminogen

**FIGURE 8:**
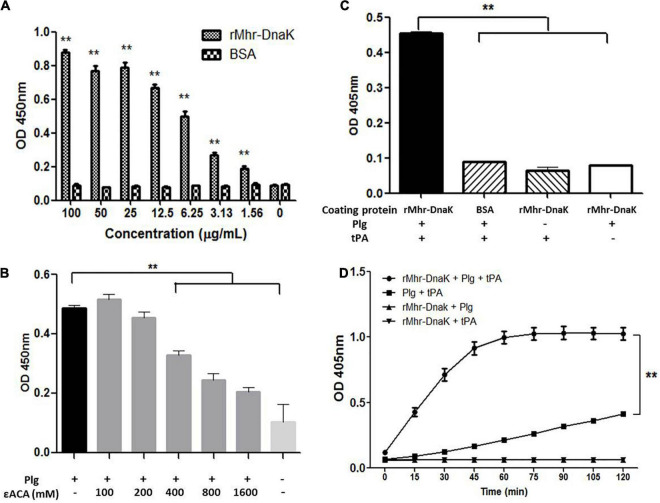
Interaction between rMhr-DnaK and host plasminogen. **(A)** The ability of rMhr-DnaK to bind plasminogen was evaluated by detecting the adherence of rMhr-DnaK to plasminogen-coated ELISA plates. BSA served as negative control. **(B)** The binding of plasminogen to the rMhr-DnaK-coated ELISA plates was inhibited by the addition of ε-ACA. **(C)** The activation of rMhr-DnaK-bound plasminogen was determined by adding tissue-type plasminogen activator (tPA) followed by plasmin-specific substrate. **(D)** The influence of rMhr-DnaK on the activation of plasminogen by tPA was further determined by detecting the activation in a solution with or without rMhr-DnaK. The assays were performed in three independent experiments. Data are the mean ± SD of triplicate wells from a representative of three independent experiments. **P < 0.01; Plg, plasminogen.

The ability of rMhr-DnaK to bind plasminogen was revealed by the microtiter plate test (*P* < 0.01, Figure 8A). The binding was significantly down-regulated by adding a lysine analog, ε-ACA (*P* < 0.01, Figure 8B). With the increase of the concentration of ε-ACA, the amount of plasminogen bound to rMhr-DnaK decreased gradually. The ability of rMhr-DnaK to capture plasminogen from normal pig plasma was demonstrated in Supplementary Figure S2. Subsequently, we determined whether the rMhr-DnaK-bound plasminogen was activated by the host agonist to form plasmin. tPA was added to activate the rMhr-DnaK-bound plasminogen. Thereafter, a plasmin-specific chromogenic substrate was added to detect their hydrolytic activity. As shown in Figure 8C, an increased OD_405*nm*_ was detected in the wells coated with rMhr-DnaK with the addition of plasminogen and tPA (*P* < 0.01) but not in the BSA-coated control wells. No activation was observed in the rMhr-DnaK-coated wells without tPA or plasminogen. To further determine whether the binding of rMhr-DnaK proteins increases the susceptibility of plasminogen to be activated by tPA, the activation of plasminogen was measured in a solution with or without rMhr-DnaK protein. As shown in Figure 8D, plasminogen was activated more rapidly in the wells containing rMhr-DnaK, plasminogen, and tPA than in wells that contained only plasminogen and tPA (*P* < 0.01). No activation was observed in the absence of tPA or plasminogen.

### DnaK-Specific Antibody Production After Immunization With Inactivated *Mycoplasma hyorhinis* Vaccine

**FIGURE 9:**
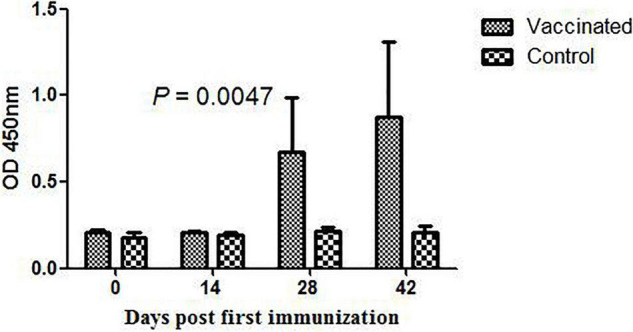
DnaK-specific serum IgG antibody in pigs immunized with the inactivated *Mycoplasma hyorhinis* vaccine. Pigs were inoculated with an inactivated *M. hyorhinis* vaccine twice with a 2-week interval. Serum samples were collected every 14 days and detected for IgG antibodies in 96-well ELISA plates coated with the rMhr-DnaK protein. Data are expressed as mean ± SD.

To assess the ability of DnaK of *M. hyorhinis* to induce a specific immune reaction, the DnaK-specific antibody in the serum of pigs inoculated with inactivated *M. hyorhinis* vaccine was determined using ELISA. As shown in Figure 9, the production of serum IgG antibody against DnaK was detected at 14 days after the second immunization, and it increased thereafter (*P* < 0.05). No antibody response was detected in the control group.

## Discussion

The phenomenon of moonlighting was first identified in GAPDH of group A streptococci (Pancholi and Fischetti, 1992) and has since been widely described in a variety of bacteria. Commonly recognized moonlighting proteins include metabolic enzymes, molecular chaperones, and protein-folding catalysts (Henderson, 2014). Usually, the moonlighting activity is dependent on the location of the protein. For example, a protein has one originally defined activity in the cytoplasm and another biological activity when it is present on the cell surface. The present study is the first to our knowledge to identify the surface localization of DnaK in *M. hyorhinis*, which indicates that it has additional functions apart from the molecular chaperoning function. DnaK does not contain a signal sequence. Therefore, it would probably be secreted through a non-classical secretion pathway and then anchored to bacterial surface. Although it has been suggested that moonlighting proteins could become released from dead or damaged cells, more evidence support that some secretion systems are need (Ebner et al., 2015, 2016; Jeffery C., 2018). It have been reported that an internal hydrophobic helical domain is essential but not sufficient for export of *Bacillus subtilis* enolase (Yang et al., 2014). Lys341 of *E. coli*, *Enterococcus faecalis* and *Bacillus subtilis* enolase is spontaneously modified with the substrate 2-phosphoglycerate, which is required for export (Boel et al., 2004). In the present study, the time course of the surface expression of *M. hyorhinis* DnaK was investigated (Supplementary Figure S3). A small amount of DnaK started to be expressed on the bacterial surface at 6 h in the logarithmic growth phase. More significant surface expression was detected at 12 h when bacterial titer reached the peak. The data indicated that the surface DnaK of *M. hyorhinis* may not be released from dead mycoplasmas.

Increasing number of moonlighting proteins have been identified to be involved in bacterial pathogenesis, by acting as surface adhesins and/or secreted signaling agents (Henderson and Martin, 2013; Jeffery C., 2018). We compared the amount of DnaK on the cell surface of *M. hyorhinis* strains with different virulence. Results showed that the high-virulence strain HEF-16 and strain JS-15 with the ability to induce pleuritis, pericarditis, peritonitis and arthritis expressed more DnaK on their surface than the avirulent strain HUB-1, which is unable to induce any diseases (Figure 3). The data suggest that DnaK may play an important role in *M. hyorhinis* virulence. However, more strains are needed to draw a final conclusion. Although the mechanism of how this chaperone protein anchors to bacterial surface remains unclear, the moonlighting functions were primarily investigated in the present study. A growing number of proteins have been reported to have not just one moonlighting activity but multiple such activities (Henderson and Martin, 2013). Likewise, the DnaK of *M. hyorhinis* was shown to have the capabilities to adhere to host cells and bind plasminogen and various ECM components.

Adherence of bacterial cells to host cell surface is often the first step in the establishment of bacterial disease. The adherence of *M. hyorhinis* to porcine PK-15 (Xiong et al., 2016) and human NCI-H292 (Supplementary Figure S4) cells has been confirmed. The adherence were significantly inhibited by anti-DnaK serum (Figure 4), but the inhibition was only partial due to the existence of other adhesins. The ability of rMhr-DnaK to bind PK-15 and NCI-H292 cells were further proved by the indirect immunofluorescence assay and MPAA (Figures 5, 6). Moreover, ECM proteins distributed around host cells are often used by pathogens as bridges to enhance their adherence to the host, which has been described in many mycoplasma species, such as *M. penumoniae* (Hagemann et al., 2017; Grimmer and Dumke, 2019), *M. hyopneumoniae* (Yu et al., 2018), *M. gallisepticum* (Furnkranz et al., 2013), and *M. fermentans* (Yavlovich and Rottem, 2007). In the present study, the ability of *M. hyorhinis* to bind fibronectin, collagen type IV, laminin, and vitronectin were detected (Supplementary Figure S5). After that, we demonstrated the interaction between rMhr-DnaK and these ECM components (Figure 7). This multimolecular interaction likely facilitates the tight adherence of *M. hyorhinis* to host cells.

The invasion of host cells by *M. hyorhinis* has been reported previously (Chernov et al., 2015; Kim B. W. et al., 2019). The niche of intracellular survival is conducive for *M. hyorhinis* to avoid recognitions by immune system components such as antibody and complement so as to achieve immune escape and long-term infection. Moreover, because several antibiotics can not effectively penetrate the cell membrane, *M. hyorhinis* in the host cell may effectively avoid being eliminated by antibiotics. Furthermore, intracellular antibiotics at subinhibitory concentrations may also promote the rapid development of drug resistance of *M. hyorhinis*, which has been reported by various clinical studies (Beko et al., 2019). The mechanism of invasion of eukaryotic cells by *M. hyorhinis* remains unclear. The interaction between bacteria and some ECM components has been reported to be related to cell invasion. Host fibronectin and vitronectin, found to interact with *M. hyrorhinis* DnaK in the present study, is related to host cell invasion by bacteria. Fibronectin and vitronectin contain the Arg-Gly-Asp (RGD) sequence for binding the integrin receptors on cell membrane, functioning as bridge between bacteria and epithelial cells. Once bacteria are attached to the fibronectin/vitronectin-integrin complex, signaling for actin remodeling in host cells is stimulated, and internalization is promoted (Scibelli et al., 2007; Singh et al., 2010).

Plasminogen has been described as the binding target of many microbial surface molecules (Raymond and Djordjevic, 2015). Plasminogen plays a key role in fibrinolysis (Law et al., 2013). Fibrin not only seals off leaking blood vessels but also helps the host to encapsulate and prevent further spread of bacterial infections. Plasmin, derived from its inactive proenzyme plasminogen, is a serine protease that degrades fibrin and various proteins of the ECM barrier, such as laminin and fibronectin (Peetermans et al., 2016). It also activates matrix metalloproteinase and further enhances the degradation of ECM components such as collagen (Lahteenmaki et al., 2005). Bacterial pathogens have developed various ways to employ the host’s plasminogen/plasmin system to promote their dissemination through tissue barriers (Raymond and Djordjevic, 2015). A few bacterial species such as staphylococci and *Yersinia pestis* produce specific plasminogen activators to hijack host’s plasminogen/plasmin system. But most pathogens bind plasminogen to their surface *via* surface plasminogen receptors (PlgRs) (Bhattacharya et al., 2012). The systemic dissemination of *M. hyorhinis* is assumed to be an important step in the occurrence of clinical diseases (Zimmerman et al., 2019). *M. hyorhnis* is able to capture plasminogen, which can be activated by tPA on its surface (Supplementary Figures S5, S6). Hijacking and abusing the plasminogen/plasmin system may play a role in the process of systemic spread of *M. hyorhini*s. One PlgR of *M. hyorhinis*, GAPDH, has been identified previously, which is a key glycolytic enzyme and also a moonlighting protein functioning both in the cytoplasm and on the surface of *M. hyorhinis* (Wang et al., 2021). In the present study, we demonstrated the ability of rMhr-DnaK to bind plasminogen (Figure 8A). The interaction was significantly down-regulated by the lysine analog ε-ACA (Figure 8B), which indicates the critical role of lysine residues in the interaction. rMhr-DnaK-bound plasminogen was activated by tPA. No activation was observed in the absence of tPA, which indicates the inability of rMhr-DnaK to activate plasminogen independently (Figures 8C,D). In addition to simply being a bridge between bacteria and plasminogen, the binding of PlgR to the lysine-binding sites in the plasminogen kringle domains facilitates the activation of plasminogen to plasmin and protects the resulting plasmin from inactivation (Bhattacharya et al., 2012; Seymour et al., 2012; Figueiredo et al., 2015). As our data shows, the activation of plasminogen by tPA was significantly enhanced by the addition of rMhr-DnaK (Figure 8D). These results suggest that DnaK is another PlgR of *M. hyorhinis*.

The complement system, which includes three different pathways, serves as the first line of immune defense. The three pathways lead to a common lytic pathway with the formation of membrane attack complex (MAC) (Merle et al., 2015). Many bacteria escape complement-mediated killing by recruiting complement regulators to their surface (Fraga et al., 2016; Krukonis and Thomson, 2020; Riesbeck, 2020). Vitronectin, a component of ECM, is also known to be a complement regulator, which inhibits C5b-7 complex formation and C9 polymerization (Milis et al., 1993). By recruiting vitronectin to surface proteins, bacteria inhibit the MAC formation and protect themselves from MAC-mediated lysis (Singh et al., 2010). Plasminogen/plasmin is another negative regulator of the complement system. It binds C3, C3b, C3d, and C5 and directly cleaves C3b and C5. Cleavage of C3b and C5 renders them non-functional and inhibits the activity of the complement system (Raymond and Djordjevic, 2015; Peetermans et al., 2016). Therefore, the interaction mediated by surface DnaK with those complement inhibitors may facilitate the escape of *M. hyorhinis* from the complement-mediated innate immune clearance.

Gene knockout is a best way to identify the function of a specific gene. But the genetic operating system is still poorly studied for most of the mycoplasma species. In addition, DnaK plays an important role in bacterial growth and reproduction, which are predicted to be essential gene in *M. hyorhinis* (Trueeb et al., 2019). Therefore, *M. hyorhinis* mutant missing DnaK gene seems to be unavailable. In the present study, antibody inhibition assay by DnaK-specific antibody was used to study the role of DnaK in the cytoadherence of *M. hyorhinis*. A systems for inducible repression of gene expression based on clustered regularly interspaced short palindromic repeats-mediated interference (CRISPRi) in *M. pneumoniae* and synthetic *M. mycoides* has been reported (Mariscal et al., 2018). This system would benefit the functional studies of the essential genes if it could be established in *M. hyorhinis* in the future.

The surface location and the multiple functions of DnaK makes it a potential candidate for developing subunit vaccines against *M. hyorhinis* infection. Previously, DnaK-based vaccine development has been reported in different species such as *Salmonella typhimurium, Salmonella typhi*, and *Mycobacterium tuberculosis* (Paliwal et al., 2011; Chuang et al., 2018; Verma et al., 2021). However, in a study on *M. pneumoniae*, no specific antibodies to DnaK were detected after infection, indicating the poor immunogenic of the protein (Hagemann et al., 2017). Similar results implying poor immunogenicity was also found in some other surface-displayed glycolytic enzymes of *M. pneumoniae* (Grundel et al., 2015). In contrast, significant IgG antibody against *M. hyorhinis* DnaK was detected in the serum of pigs immunized with inactivated *M. hyorhinis* in the present study (Figure 9). The finding indicates good exposure and immunogenicity of *M. hyorhinis* DnaK and supports its potential use in subunit vaccine development. However, the presence of antibody does not indicate that these antibodies are protective. It needs more experiment to investigate whether DnaK is one of the key protective antigens of *M. hyorhinis*.

## Conclusion

Taken together, the present study identified the surface exposure of DnaK in *M. hyorhinis*. Its ability to adhere to host cells, bind different ECM components, and bind to and enhance the activation of plasminogen was investigated. By interacting with those host molecules, DnaK may moonlight in various important pathogenic processes of *M. hyorhinis*, such as adhesion, cell invasion, systemic dissemination, and evasion of immune response.

## Data Availability Statement

The original contributions presented in the study are included in the article/Supplementary Material, further inquiries can be directed to the corresponding authors.

## Ethics Statement

The animal experimental procedures conformed to the guidelines of Jiangsu Province Animal Regulations (Government Decree No. 45). The animals in this study were under ethical approval by the Committee on the Ethics of Animal Experiments in Jiangsu Academy of Agricultural Sciences (Protocol# PDC 2021006 and Protocol# PDC 2021011). All efforts were made to minimize animal suffering in animal experiments.

## Author Contributions

YL and JW completed the study of the pathogenic mechanism of DnaK and prepared the manuscript. BL and YW helped with the cell adhesion experiments. YY did the interaction analysis between rMhr-DnaK and plasminogen. TY and YG performed the animal experiments. JS helped to prepare the recombinant protein. ZF, ZT, and GS modified the manuscript. QX supervised and guided this work. All authors read and approved the final manuscript.

## Conflict of Interest

The authors declare that the research was conducted in the absence of any commercial or financial relationships that could be construed as a potential conflict of interest.

## Publisher’s Note

All claims expressed in this article are solely those of the authors and do not necessarily represent those of their affiliated organizations, or those of the publisher, the editors and the reviewers. Any product that may be evaluated in this article, or claim that may be made by its manufacturer, is not guaranteed or endorsed by the publisher.
