# Supplementary material for: DnaK Functions as a Moonlighting Protein on the Surface of Mycoplasma hyorhinis Cells
Source: Front Microbiol. 2022 Mar 3;13:842058. doi: 10.3389/fmicb.2022.842058 (PMC8927758; doi:10.3389/fmicb.2022.842058)
Supplement: Supplementary file 1 [file Data_Sheet_1.docx]

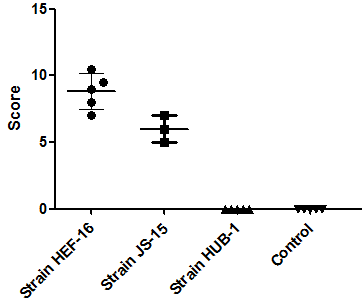


**Supplementary Fig. S1.** **Virulence evaluation of the *M. hyorhinis* strains by *in vivo* challenge test.** Pigs were inoculated with 10^10^ CCU of different *M. hyorhinis* strains via a combined route (intravenous + intraperitoneal + intranasal). Twenty-one days after the challenge, all the animals were slaughtered. Pleuritis, pericarditis, peritonitis and arthritis were scored and a total score was calculated as the sum of the score for the four tissues.


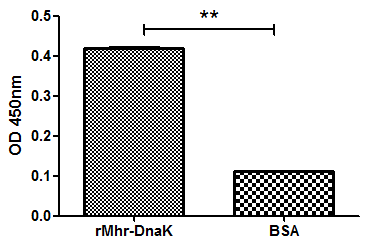


**Supplementary Fig. S2. Ability of rMhr-DnaK to bind plasminogen from normal pig plasma.** ELISA plate was coated with rMhr-DnaK or BSA. After blocking, pig plasma was added. The bound plasminogen was detected by rabbit anti-plasminogen polyclonal antibody followed by HRP-conjugated goat anti-rabbit IgG.


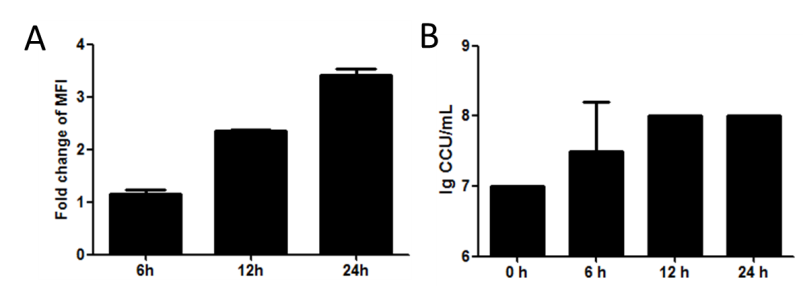


**Supplementary Fig. S3.** **Surface expression of DnaK in *M. hyorhinis* at different growth stages detected by FACS.** *M. hyorhinis* cells were harvested from cultures of 6 h, 12 h, 24 h, respectively. Then the pellets were washed twice with PBS before incubating with rabbit anti-DnaK serum or preimmune serum. The FITC-labeled goat anti-rabbit IgG antibody was used as secondary antibody. The fluorescence of mycoplasmal cells was detected using a BD Accuri C6 flow cytometer. The mean fluorescence intensity (MFI) was expressed as the percentage of that of *M. hyorhinis* incubated with preimmune serum. CCU of the samples were detected.


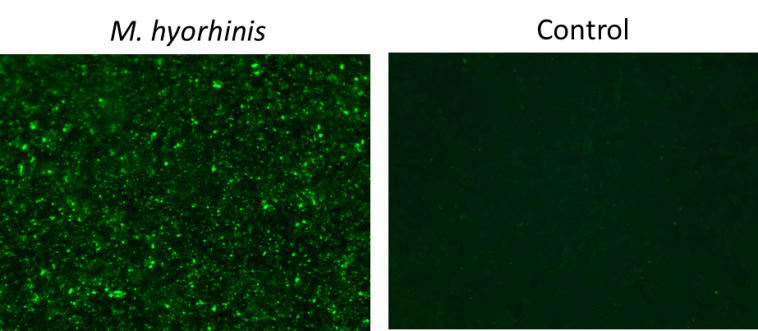


**Supplementary Fig. S4. Adherence of *M. hyorhinis* to human NCI-H292 cells detected by indirect immunofluorescence assay.** NCI-H292 cells were incubated with *M. hyorhinis* or PBS. Bound *M. hyorhinis* were detected by anti-P37 polyclonal antibody and FITC-labeled goat anti-rabbit IgG.


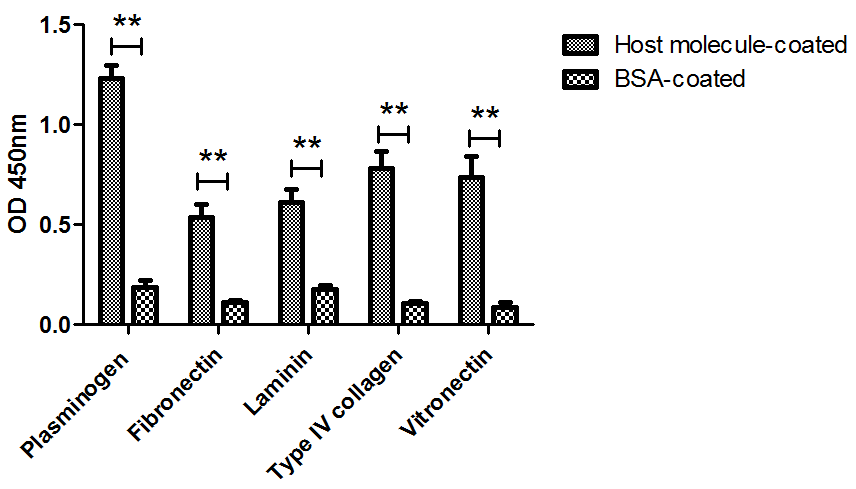


**Supplementary Fig. S5. Ability of *M. hyorhinis* to bind plasminogen and ECM components.** *M.* *hyorhinis* cells were collected by centrifugation and blocked with PBS contain 5% BSA. After that, *M. hyorhinis* cells were added into ELISA plate coated with plasminogen and different ECM components. The bound *M. hyorhinis* cells were detected by anti-P37 polyclonal antibody followed by HRP-conjugated goat anti-rabbit IgG.


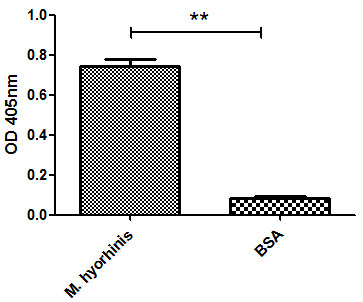


**Supplementary Fig. S6. Activation of plasminogen bound to *M. hyorhinis* by tPA.** *M. hyorhinis* cells were collected by centrifugation and used to coat ELISA plates. After blocking, plasminogen was added and incubated. After washing, tPA was added and followed by substrate. OD_405nm_ was measured after overnight incubation.

**Supplementary Table S1. Evaluation of the demage to *M. hyorhinis* before the binding of anti-DnaK antibody.** *M. hyorhinis* cultures were centrifuged at 15,000 × *g* for 20 min at 4°C. The cells in the precipitate were blocked with TBS containing 5% skim milk or PBS containing 1% BSA. *M. hyorhinis* cells were collected and resuspended with the same volume of fresh KM2 medium. The CCU of *M. hyorhinis* before and after treatment was detected.

| *M. hyorhinis* | CCU |
| --- | --- |
| Before blocking | 10^8^ CCU/mL |
| After blocking with TBS containing 5% skim milk | 10^8^ CCU/mL |
| After blocking with PBS containing 1% BSA | 10^8^ CCU/mL |
